# First identification of potato tuber rot caused by *Penicillium solitum*, its silver nanoparticles synthesis, characterization and use against harmful pathogens

**DOI:** 10.3389/fpls.2023.1255480

**Published:** 2023-10-19

**Authors:** Syed Haseeb Shah, Xiaoliang Shan, Sofia Baig, Hongwei Zhao, Bushra Ismail, Irum Shahzadi, Zahid Majeed, Shamyla Nawazish, Maria Siddique, Ayesha Baig

**Affiliations:** ^1^ Department of Biotechnology, COMSATS University Islamabad, Abbottabad, Pakistan; ^2^ Department of Plant Pathology, College of Plant Protection, Nanjing Agricultural University, Nanjing, China; ^3^ Independent Researcher, Abbottabad, Pakistan; ^4^ Department of Chemistry, COMSATS University Islamabad, Abbottabad, Pakistan; ^5^ Department of Biotechnology, The University of Azad Jammu and Kashmir, Muzaffarabad, Pakistan; ^6^ Department of Environmental Sciences, COMSATS University Islamabad, Abbottabad, Pakistan

**Keywords:** *Penicillium solitum*, silver nanoparticles, potato, antifungal, antibacterial

## Abstract

Potato is one of the highly consumed vegetable crop grown in different regions across Pakistan that is affected by fungal diseases. The current research was conducted to identify fungal pathogen causing mold-like disease of potato in Khyber Pakhtunkhwa (KP), Pakistan. For molecular identification and characterization of the fungal disease; potato tuber samples were collected followed by culturing on potato dextrose agar (PDA). Based on morphological features, the pathogen was identified as a *Penicillium* species. This result was obtained in 45 different isolates from potato tubers. Molecular identification was done using β-tubulin primers and ITS5 sequencing of 13 different isolates that releveled 98% homology with BLAST (GenBank accession no. KX958076) as *Penicillium solitum* (GenBank accession nos. ON307317; ON307475 and ON310801). Phylogenetic tree was constructed that showed *Penicillium solitum* prevalence along with *Penicillium polonicum* and *Penicillium citrinum* on potato tubers. Based on this, *Penicillium solitum* based silver nanoparticles (Ag NPs) were synthesized and characterized using UV-visible spectroscopy, Fourier transform infrared (FTIR) spectroscopy, X-ray diffraction (XRD), energy dispersive X-ray (EDX) and field emission scanning electron microscopy (FE SEM). UV-analysis showed a characteristic peak at 410 nm confirming synthesis of *Penicillium solitum* based Ag NPs. This was further confirmed by XRD followed by EDX and SEM that showed face cubic crystal structure with Ag as major constituent of 18 nm formed spherical Ag NPs. FTIR showed band stretching of O-H, N-O and C-H of biological origin. Similarly, *Penicillium solitum* based Ag NPs presented strong anti-bacterial and anti-fungal activity at 0.5 level of significance LSD. According to our knowledge, this is the first report of *Penicillium solitum* identification in Pakistan, its Ag NPs synthesis and characterization to be used against pathogens of agricultural significance.

## 1 Introduction

Potato belongs to family *Solanaceae* that is a highly valued vegetable crop due to its role in food and nutrition. In Pakistan, potato is mainly grown in Punjab followed by KP, Sindh, Gilgit Baltistan and Balochistan province (Majeed and Muhammad, 2018). The temperate climate of KP is well suited for potato production, however its growth is frequently affected by fungal diseases (Abbas et al., 2013). Penicillium; a fungal pathogen that belongs to the genus ascomycetes, causes blue mold on many edible items like cereals, grains, corn, fruits and vegetables (Kim et al., 2006). This genus includes almost 300 species with some involved in the production of important secondary metabolites (Larsen et al., 2007). Morphological characters includes fungal mycelia having uni or multinucleated hyphae separated by a septum. Conidia are borne on conidiophores, which are round and uninucleate (Kim et al., 2007). *Penicillium solitum* is an asexually reproducing fungus in the genus *Penicillium.* It is a mesophilic and psychrotolerant species. *Penicillium solitum* produces compounds such as polygalacturonase involved in cell wall break down; solistatinol and viridicatin, polyketide-derived compactin used as cholesterol-lowering compounds generally known as statin (Larsen et al., 2007). *Penicillium solitum* initially produces white colony that later turn dark blueish green on Czaek yeast extract and PDA media while it produces brownish orange color on malt extract agar. Brown-orange color on malt extract media is unique to *Penicillium solitum* species. *Peculium solitum* has been isolated from Saffron Corm in China and similarly Blue Mold on Apple Caused by *Penicillium polonicum* in the United States has been reported (Bradshaw et al., 2021; Zhang et al., 2020). To our knowledge this is the first report of blue mold caused by *Penicillium solitum* on potato in Pakistan.

One of the modern researches in the field of nanotechnology is the production of nanoparticles of 1-100 nm in size that have significant applications in health, agriculture and medicine (Thul et al., 2013). Physical methods for nanoparticles synthesis such as thermal, plasma, ultrasonic irradiation, and laser ablation are costly and require extensive energy input for their production. On the other hand, chemical methods do not require as much energy, however these methods involves toxic chemicals that are considered environmentally hazardous and produce harmful by-products (Ganachari et al., 2012; Yassin et al., 2021). Biological based nanoparticles are environmentally friendly, cost-effective and follow relatively simple procedures that can serve as an alternative to chemical and physical methods. The biological approach based on algae, fungi, bacteria and plants follows the principle of green chemistry during the synthesis of nanoparticles (Adil et al., 2015) such as Ag NPs. Microorganisms such as bacteria, fungi, yeasts, and algae have been known to synthesize Ag NPs, but fungi are preferred for their large biomass and synthesis of some novel extracellular metabolites (Guilger-Casagrande and de Lima, 2019). Secondary metabolites such as alkaloids, terpenoids, organic acids, polyketides, polyphenolics and cyclodepsipeptides are reported in *Penicillium* genus. These bioactive compounds enhance electron donor and free radical scavenging ability resulting in capping and stabilization of nanoparticles (Rudrappa et al., 2023). Ag NPs synthesis based on *Penicillium verrucosum*, *Penicillium citrinum* and *Aspergillus niger* are reported (Yassin et al., 2017; Al-Zubaidi et al., 2019; Yassin et al., 2021). Green synthesized Ag NPs using *Penicillium oxalicum* showed cytotoxic potential against the breast cancer cell lines. Anticancer activity was noted by decreased wound closer of cancer cells and differential genes expression in tumor suppression, cell cycle arrest and Caspase-3 induced apoptosis (Gupta et al., 2023). These Ag Nps showed antimicrobial activity against *Aspergillus flavus*, *Aspergillus niger*, *Penicillium albicans* and *Staphylococcus aureus*. Similarly, *Penicillium chrysogenum* Ag NPs showed anti biofilm activity against opportunistic pathogenic *Acinetobacter baumannii* prevalent in hospital settings (Barabadi et al., 2022). In another study *Penicillium verhagenii* selenium nanoparticles were found most efficient for their antioxidant and anti-cancer activity. Additionally, these green selenium NPs were effective against larval instar mosquito *Aedes albopictus* (Nassar et al., 2023). Based on the immense importance of *Penicillium* species and wide application of Ag NPs, the current study is aimed at the identification and characterization of *Penicillium solitum* from Pakistan for the first time and its Ag NPs synthesis and characterization using UV, XRD, SEM, FTIR and EDX for their potential use against some harmful microorganisms.

## 2 Materials and methods

### 2.1 Sample collection and isolation of *Penicillium solitum* from diseased tubers

Diseased potato tuber samples having greenish-brown fungal infection were collected from different regions of KP Pakistan. The area included Abbottabad, Mansehra, Batakundi and Haripur. Samples that showed distinct mold like symptoms led us to collect potato tubers mostly from the north of Pakistan for its molecular identification and characterization.

For fungus isolation, infected potato tubers were washed with 70% ethanol for 15-20 seconds and later rinsed with distilled water. The infected portion of the tuber was cut and crushed using distilled water. PDA media was used for sample inoculation with serial dilution. After inoculation, the samples plates were stored at 28^0^C for 5-7 days. Samples having morphologically distinct mold like features were re-cultured on PDA medium. Re-culturing was done until homogenized colonies were obtained following the same culturing procedure.

### 2.2 PCR analysis of β-Tubulin and pathogenicity test

CTAB method was used for the extraction of DNA from different fungal cultures (Zhang et al., 2010). Briefly, fungal inoculum was taken in TE buffer, glass beads were added and subjected to vortex for 20 mins. It was then centrifuged at 10000 rpm for 15 mins. 200µl sample was taken and subjected to CTAB extraction method and DNA obtained was suspended in TE buffer. For Penicillium identification, β-Tubulin gene (Kim et al., 2007) was amplified at 57^0^C annealing temperature. Pathogenicity test was performed on certified potato tubers for symptoms development. The tubers were surface sterilized with 70% ethanol and washed with distilled water. A 50ul spore suspension of 3x 10^4^/ml in sterile distilled water was used to inoculate potato tubers. Tubers were stored at 25°C for 7 days for symptoms development.

### 2.3 Phylogenetic tree construction

**Figure 1:**
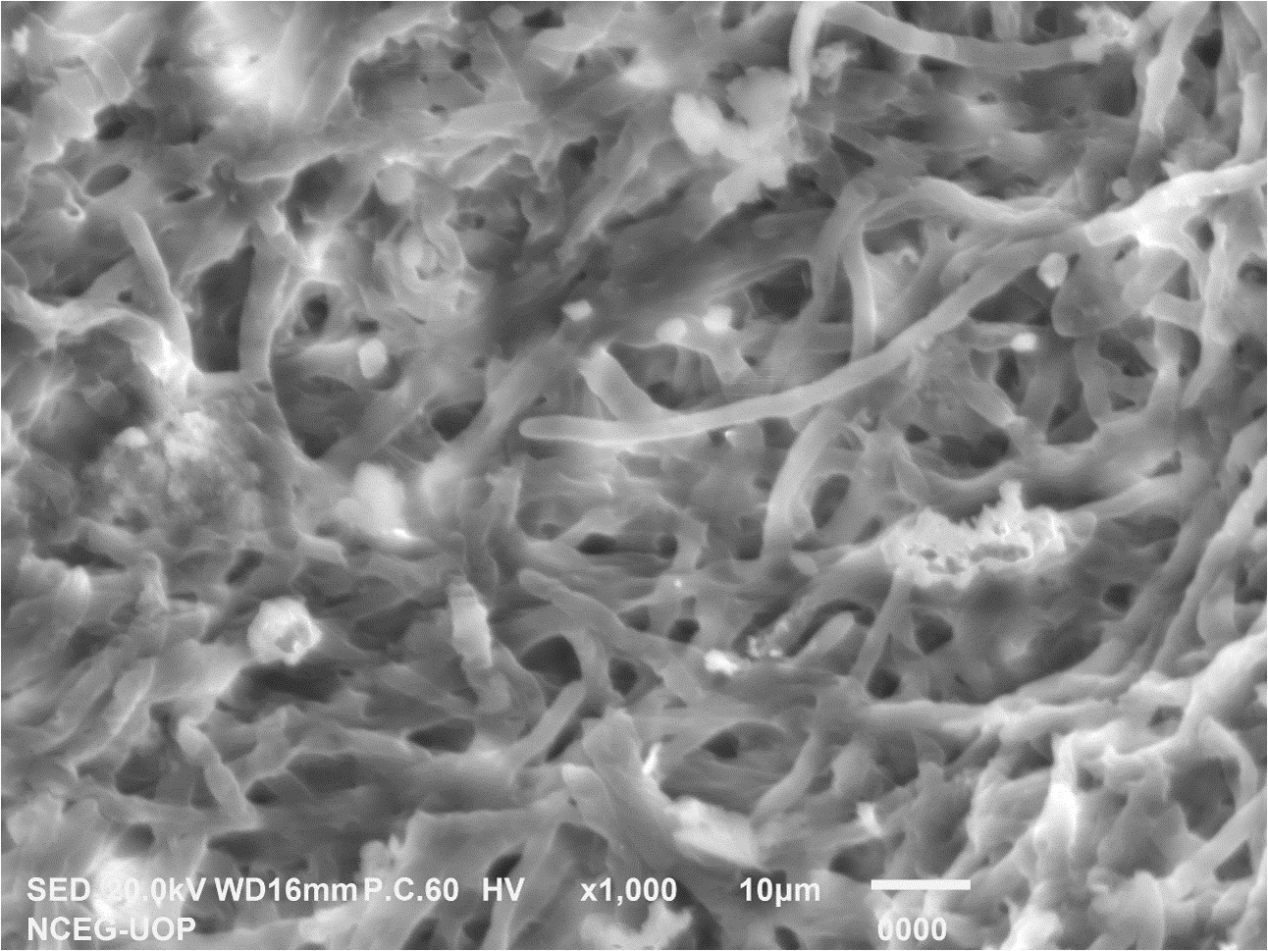
Scanning electron microscopy of *Penicillium solitum*.

We performed ITS single-end sequencing of 13 strains using Illumina sequencing technology and obtained the sequences of these strains. Newly generated 13 sequences were submitted to Gene Bank and accession numbers are provided in **Table S1**. Then, we performed Blastn analysis on these ITS sequencing results and corresponding 10 sequences were selected with 13 isolated strains to construct a phylogenetic tree (**Figure 1**). Based on the results of the pre-built phylogenetic tree, we used PhyloSuite tool (Zhang et al., 2010; Zhang et al., 2020) to align 24 sequences using MAFFT with *Penecillium glabrum* CBS 125543 as an outgroup species (Katoh and Standley, 2013). Maximum likelihood analyses were run with IQ-TREE2 (Minh et al., 2020), using models selected by ModelFinder (Kalyaanamoorthy et al., 2017); Using Shimodaira–Hasegawa–like approximate likelihood-ratio test (SH-aLRT) fast branch tests support in the ML tree (Hoang et al., 2018). The ITS phylogenetic tree includes 24 sequences, using the K2P+R2 as best-fit model.

### 2.4 Biomass preparation and synthesis of Ag NPs

The method described by (Zonooz and Salouti, 2011) was slightly modified for microbial mediated synthesis of Ag NPs. The *Penicillium solitum* strain ON307317 identified in the current research study was cultured on PD broth and incubated at 28°C and 210 rpm. After 7 days of incubation, the culture was centrifuged at 9000 rpm for 20 min and the biomass was collected. This biomass was added to 250mL of autoclaved distilled water for 3 days of incubation at 28°C. After incubation, the solution was filtered again, and this filtrate was used for the biosynthesis of Ag NPs. For Ag NPs synthesis, about 30 ml of cell-free supernatant and 20 ml silver nitrate (AgNO_3_) solution were incubated at 28°C and 150 rpm for 72 h. After the incubation period, the solution was observed for visual color change. The flasks turned from opaque white to dark brown that indicated the presence of bio synthesized Ag NPs (Zonooz and Salouti, 2011; Patil et al., 2019).

### 2.5 UV–vis spectral analysis of Ag NPs

Ag NPs synthesized by *Penicillium solitum* cell-free culture supernatant was monitored by UV–vis Spectrophotometer (UV-1602, BMS) within a range of 200–800 nm (Nayak et al., 2011).

### 2.6 FTIR spectral analysis of synthesized Ag NPs

Bio reduction of Ag^+^ ions using the cell-free culture supernatant of *Penicillium solitum* was monitored by FTIR (Thermo Scientific, USA) and the spectra were recorded at an interval between 4000 and 400 cm^−1^ (Dinesh et al., 2021; Rose et al., 2019).

### 2.7 FE SEM analysis of Ag NPs

For taking the FE SEM images of the biosynthesized Ag NPs, a drop of suspension was placed on specimen stubs. The micrographs for Ag NPs were obtained at 10kV under (5–10 torr) vacuum pressure and the presence of metallic Ag in the solution was analyzed by EDS by using MIRA3 TESCAN model electron microscope (Yousaf et al., 2022).

### 2.8 XRD measurement of *Penicillium solitum* Ag NPs

To examine the crystal structure of biosynthesized Ag NPs, XRD patterns were collected using Cu radiation at 40 kV/20 mA using continuous scanning 2*θ* mode on an X-ray diffractometer (BRUKER D8).

The average crystallite sizes of samples were calculated using Scherrer’s formula:


D= Kλ/ β1/2 cosθ

D represents size of the particle, K is constant, λ is wavelength, β is full width at half maxima and θ is Bragg’s angle. The acquired diffraction patterns were plotted by using Origin software for structural analysis of *Penicillium solitum* nanoparticles (Du et al., 2015).

### 2.9 Antimicrobial activity of Ag NPs

The agar well diffusion method was used for the antimicrobial activity of *Penicillium solitum* synthesized Ag NPs (Chavez-Esquivel et al., 2021). Fresh fungal culture of *Aspergillus flavus* AG1 was spread on PDA and bacterial cultures of *Streptomyces scabies and Pseudomonas syringe* DC3000 were spread on Nutrient agar plates for the formation of fungal and bacterial lawns. Six wells were prepared consisting of 20 µg/µL, 40 µg/µL and 60 µg/µL concentrations of *P. solitum* NPs. For positive control, Kanamycin was used against the bacterial cultures and Voriconazole was used against fungal strain. For negative control, distilled water was used. 60µL of AgNO_3_ (1mg/mL) was used in each plate. The bacterial plates were then incubated for 24 hrs. at 37°C and fungal cultures were incubated for 5 days at 28°C. Antimicrobial activity was determined by measuring the diameter of the zone of inhibition for Ag NPs activity (Shahzadi et al., 2022; Yousaf et al., 2022).

### 2.10 Statistical analysis

The data for antimicrobial activity was analyzed by R software (R Core Team, 2023). Data measurements were averaged with standard error ( ± SE). One-way analysis of variance (ANOVA) was performed at p < 0.05 using least significant difference (LSD) test.

## 3 Results and discussion

### 3.1 Isolation and PCR analysis of blue mold of potato

**Figure 2:**
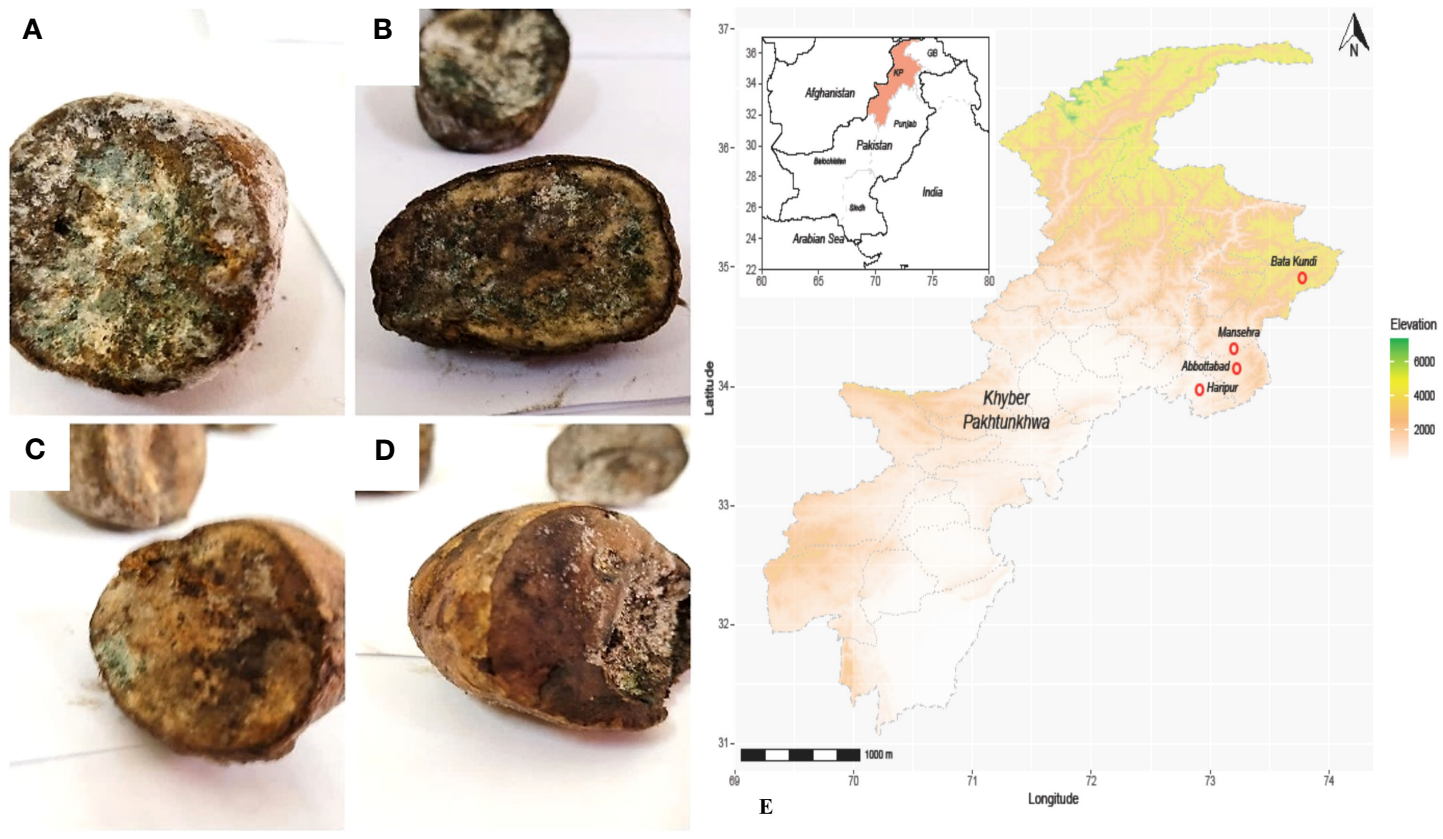
Diseased tubers from KP Pakistan; **(A)** Batakundi **(B)** Mansehra **(C)** Haripur **(D)** Abbottabad **(E)** Map of Pakistan-KP showing clear symptoms of greenish mold and rotted browning caused by Penicillium.

**Figure 3:**
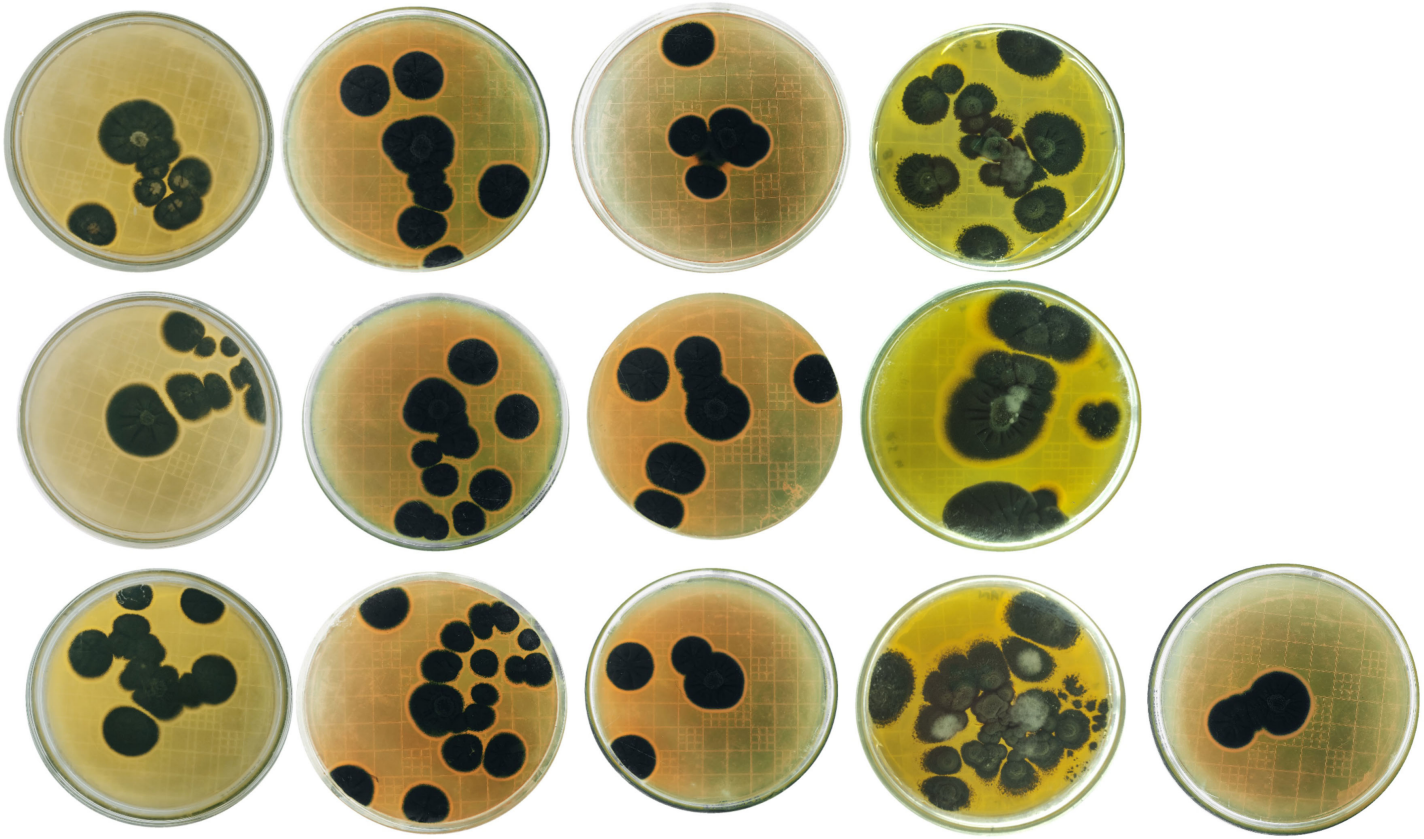
Penicillium cultures from Batakundi, Manshera, Abbottabad and Haripur, KP, Pakistan with species and accession numbers from left to right row wise *P. citrinum* ON310800 (GeneBank); *P. citrinum* ON310830 (GeneBank); *P*. *citrinum* ON310858 (GeneBank); *P. citrinum* ON310859 (Gene Bank); *P. solitum* ON307317 (GeneBank); *P. solitum* ON307475 (GeneBank); *P. citrinum* ON352773 (GeneBank) *P*. *citrinum* ON310863 (GeneBank); *P. polonicum* ON310860 (GeneBank); *P. citrinum* ON31148 *P. citrinum* ON311281 *P. citrinum* ON318976 *P. solitum* ON310801 (GeneBank database).

**Figure 4:**
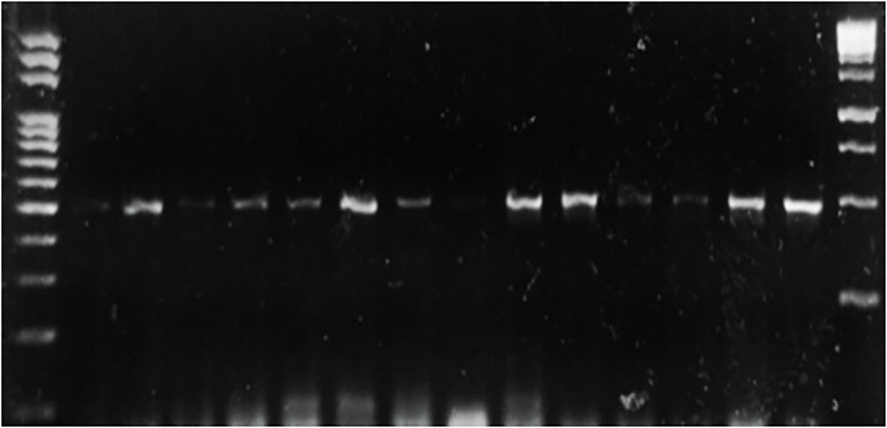
PCR of selected Penicillium isolates using β tubulin primers on DNA extracted from different potato samples showing blue mold symptoms.

Potato tubers from Batakundi, Mansehra, Abbottabad and Haripur with mold like symptoms were collected from different areas of northern KP and grown on PDA media. Cultured plates with colonies showing morphological distinct features were picked up and cultured again. These symptoms on potato tubers were never characterized that are frequently observed in potato fields of KP Pakistan. Different fields of 4 major potato growing area in KP were selected; including 8 fields in Mansehra; 3 fields in Abbottabad; 2 fields in Batakundi and Haripur each (**Figure 2**). Morphological features for the disease showed greenish black patches as in case of *Penicillium* Blue mold (Kim et al., 2006). After 7 days of incubation at 28°C fungal colonies appeared on PDA media from infected tubers (**Figure 3**). Three times plate cultures were picked up and subjected to PCR, sequencing analysis. PCR result was obtained with β-tubulin (Kim et al., 2007) identified isolates as *Penicillium* species showing approximately 550 bp band size (**Figure 4**).

### 3.2 Phylogenetic tree for *Penicillium soltium* characterization

**Figure 5:**
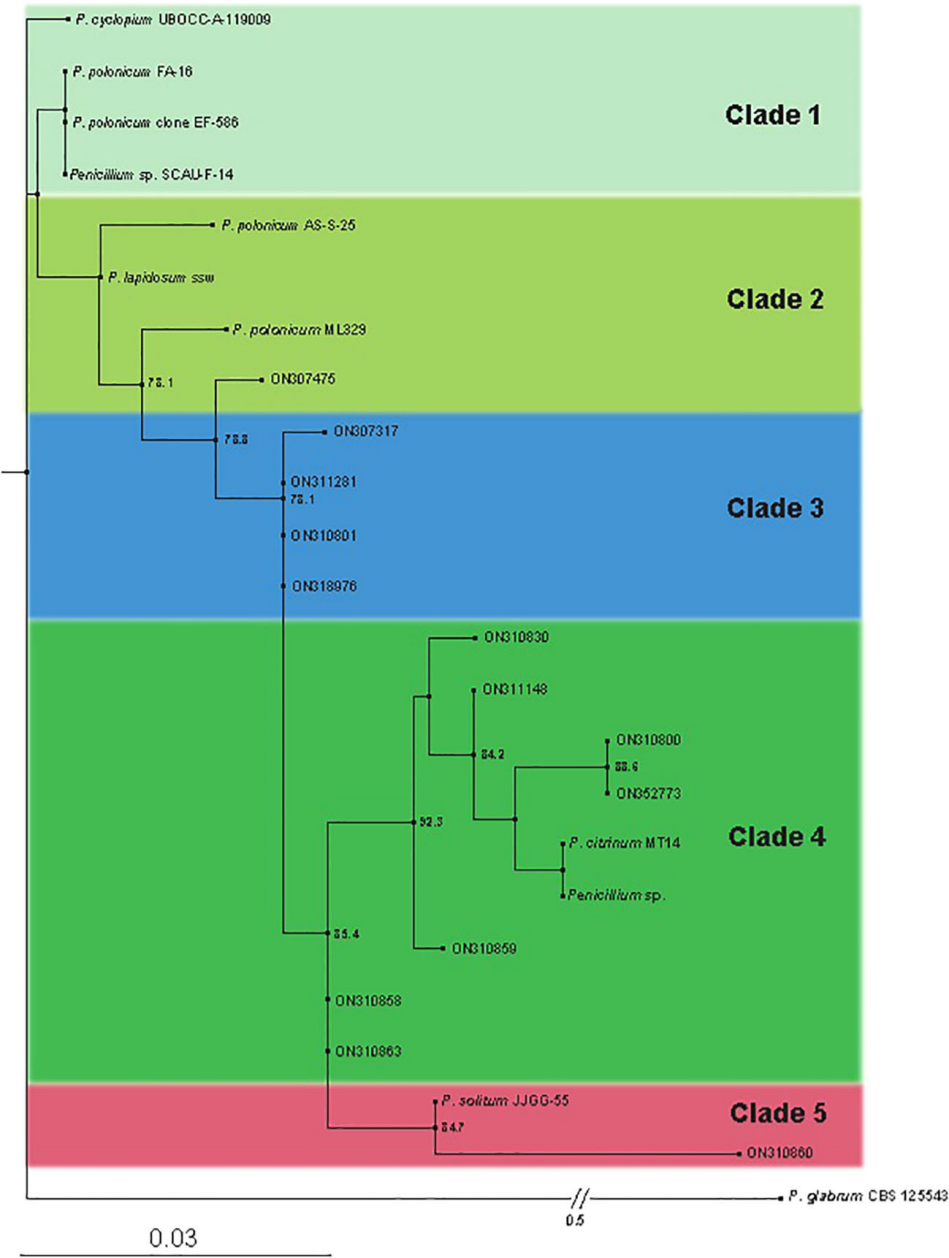
Phylogenetic tree obtained by Maximum Likelihood (ML) inferred from Internal transcribed spacer (ITS). Best-scoring ML tree obtained by IQ-tree. Only SH-aLRT support (%) values greater than 70% are shown. The names of the newly described species are in bold. Branch lengths are proportional to distance. The tree is outgroup with *P. glabrumm* CBS 125543.

The phylogenic tree constructed using ITS4 primers sequencing (Macrogen) indicated the ancestor for all of the strains as *Penecillium cyclopium* (UBOCC-A-119009)*. Penicillium citrinum* followed by *Penicillium solitum* were found more frequent in our study as closely related species of *Penicillium.* The phylogenetic consisted of 5 clades/clusters. Clade 1 consists of prime ancestor species *Penicillium cyclopium* and *Penicillium polonicum*. Clade 2 contain *Penicillium polonicum*, *Penecillium lapidosum*. Clade 3 show all the descendants of *Penicillium polonicum* and should be classified as *Penicillium polonicum*. Clade 4 is comprised of *Penicillium citrinum* only and contain more than 50% of the field-collected strains in this clade (7/13). Clade 5 consists of *Penicillium solitum* only. Isolate ON310860 is the youngest of all *Penicillium* species, which is recently differentiated and deserves further study. Species present in each clade are closely related to each other (**Figure 5**). Our study indicated *Penecillium cyclopium* as the prime ancestor of 13 *penicillium* species isolates of blue mold on potato.

### 3.3 Pathogenicity test re-created the symptoms of *Penicillium solitum*

**Figure 6:**
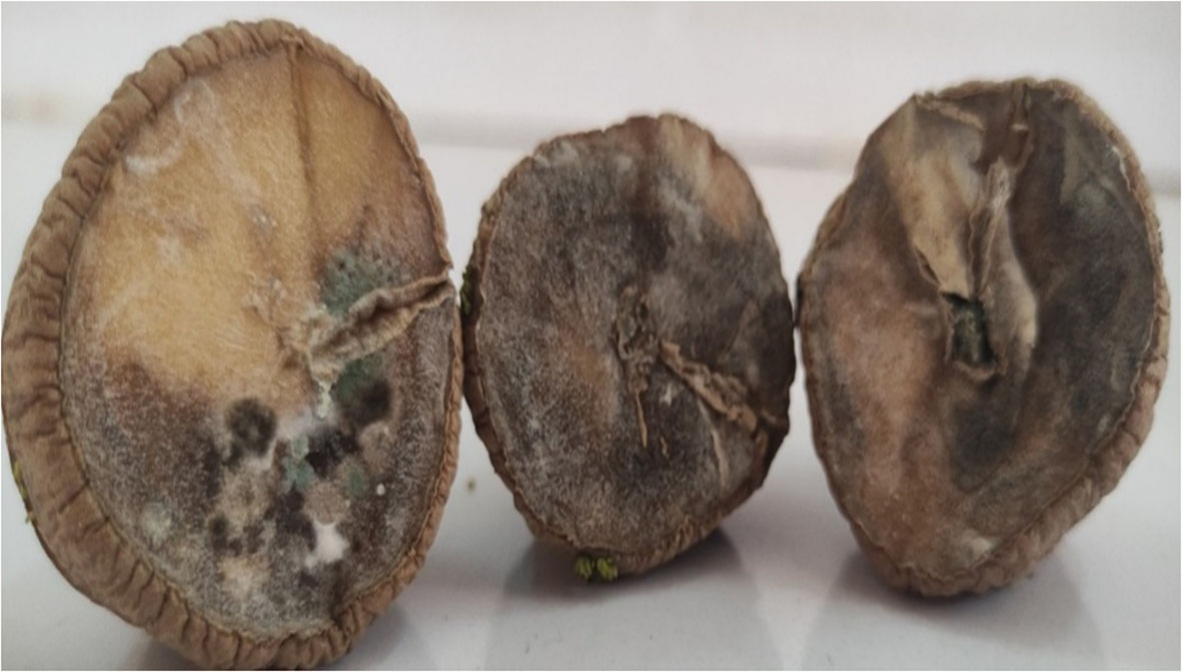
Pathogenicity test with diseased symptoms of *Penicillium solitum* on certified tubers.

To regenerate blue mold disease, potato tubers were inoculated with *Penicillium soltium* (**Figure 1**). After 7 days, disease symptoms on potato tubers appeared as blue green patches of mycelia when inoculated with *Penicillium solitum* and after 10 days, these patches transformed into black indentation with dark green patches in cross section upon dissection. The colonization pattern of *Penicillium solitum*, *Penicillium polonicum*, and *Penicillium citrinum* is almost same but in case of *Penicillium solitum*, dark green colony appeared that didn’t change its color over time whereas *Penicillium polonicum* and *Penicillium citrinum* appeared blue green or light green and changed color to dark green or black on PDA media. We therefore conclude that although *Penicillium solitum*, *Penicillium polonicum* and *Penicillium citrinum* are prevalent in potato diseased samples, however this is the first report of *Penicillium solitum* prevalence in potato field in Pakistan (**Figure 6**).

### 3.4 Preparation of *Penicillium soltium* Ag NPs

**Figure 7:**
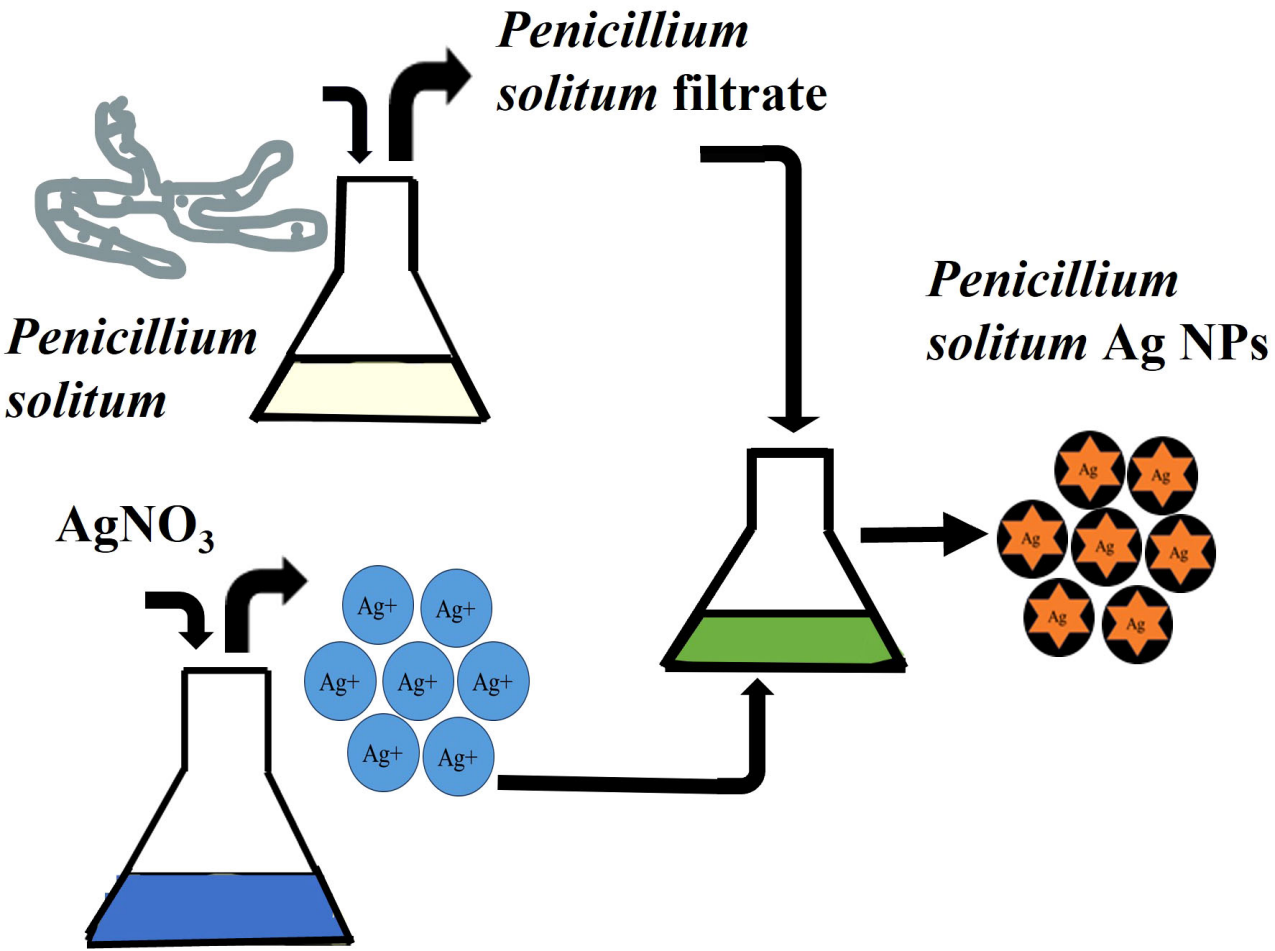
Biogenic pathway of the synthesis of Ag NPs using extracellular extract of *Penicillium solitum*.

**Figure 8:**
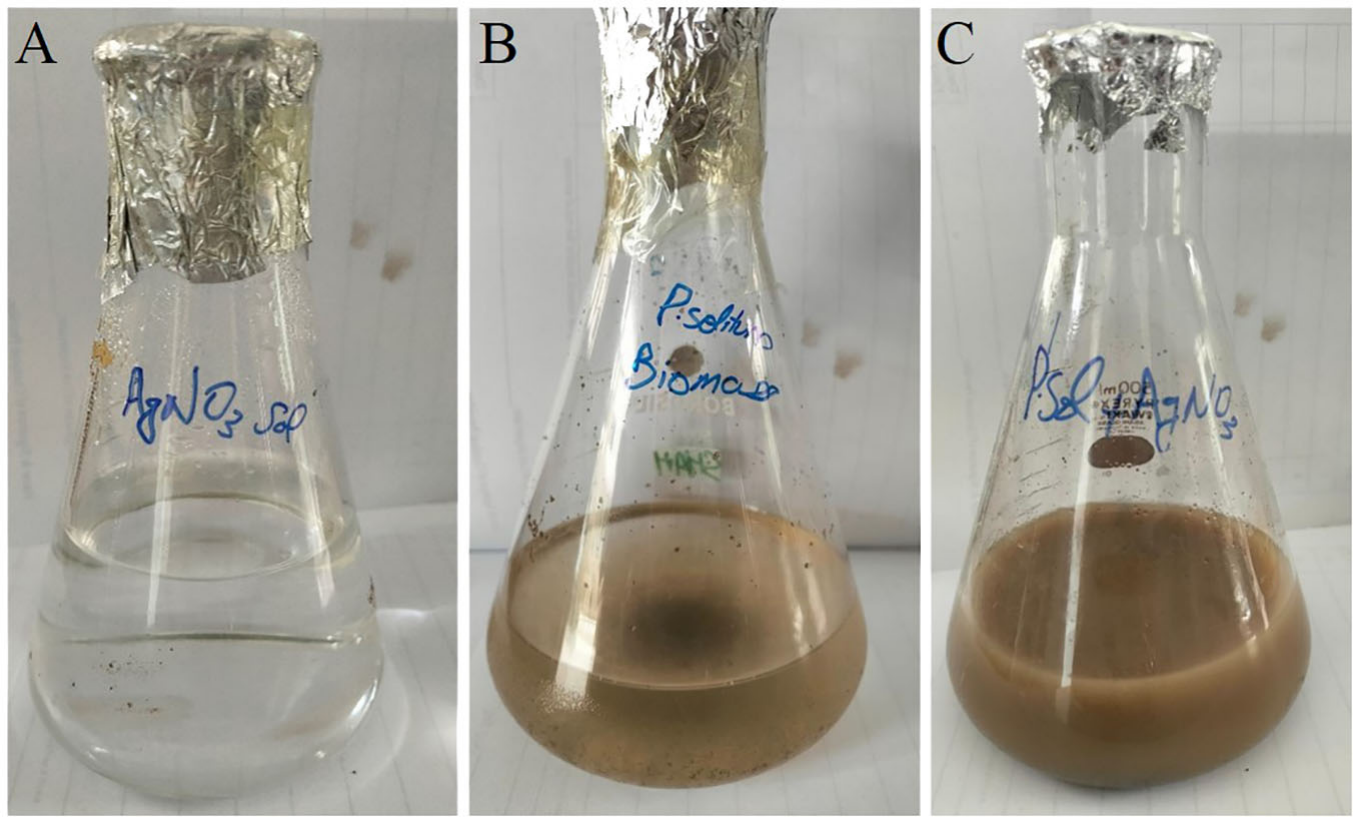
Synthesis of *Penicillium solitum* Ag NPs. **(A)** AgNO_3_ **(B)** Fungal Biomass **(C)** Ag NPs.

Once the prevalence of *penicillium solitum* was confirmed from potato growing fields in Pakistan, green Ag NPs based on *Penicillium solitum* extract were prepared. Silver nanoparticles were synthesized by stirring mixture of aqueous silver nitrate solution (1mg/mL) and *Penicillium solitum* extract in 2:3 ratio constantly at 28^0^C (Nayak et al., 2011; Yassin et al., 2021). The pH was adjusted to 11. After a while, the yellow color reaction mixture gradually changed into a dark-brown suspension (**Figures 7**, **8**). This showed that silver ions in the reaction have been transformed into silver (Niraimathi et al., 2013).

### 3.5 UV-vis spectroscopy for Ag NPs

**Figure 9:**
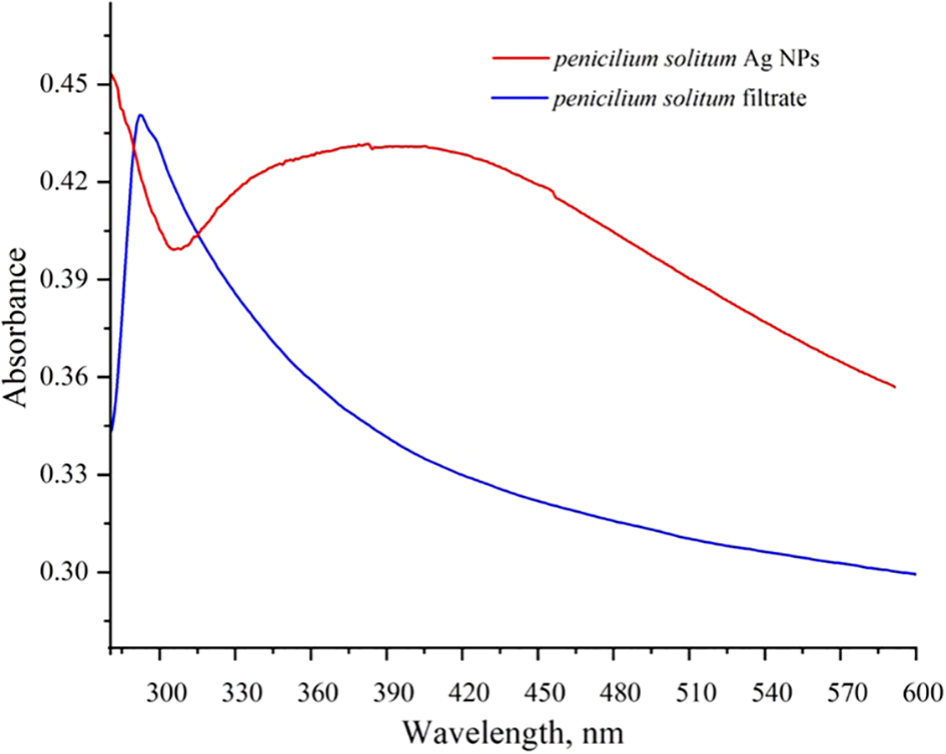
UV-Visible spectrum of *Penicillium solitum* filtrate and Ag NPs.

UV-vis spectroscopy is a primary but an important method that shows Ag NPs formation at the initial synthesis stage. Ag NPs characteristic peak depend upon the shape, size, and distribution of Ag NPs based on surface plasmon resonance (SPR). The maximum absorbance peak of *Penicillium solitum* extract was recorded at 280 nm that is attributed to aromatic structures in proteins or amino acids (**Figure 9**; Barabadi et al., 2022). The peak shifted to 410 nm that confirmed the synthesis Ag NPs (**Figure 9**; Sasidharan et al., 2020). The peak at 410 nm can be attributed to the phenomenon of SPR when the conduction electrons on the bio-fabricated Ag surface oscillate collectively in response to specific wavelength of light. The observed peak at 410 nm indicated secondary metabolites mediated reduction and capping of Ag NPs by *Penicillium solitum*. A similar characteristic peak was observed in a previous study due to SPR in Ag NPs synthesis mediated by *Penicillium oxalicum* (Gupta et al., 2023). Similarly, UV–vis spectrum of Ag NPs produced by *P. citrinum* exhibited an absorption band at around 400–420 nm, suggesting the synthesis of Ag NPs by the fungus (Yassin et al., 2017).

### 3.6 XRD of Ag NPs

**Figure 10:**
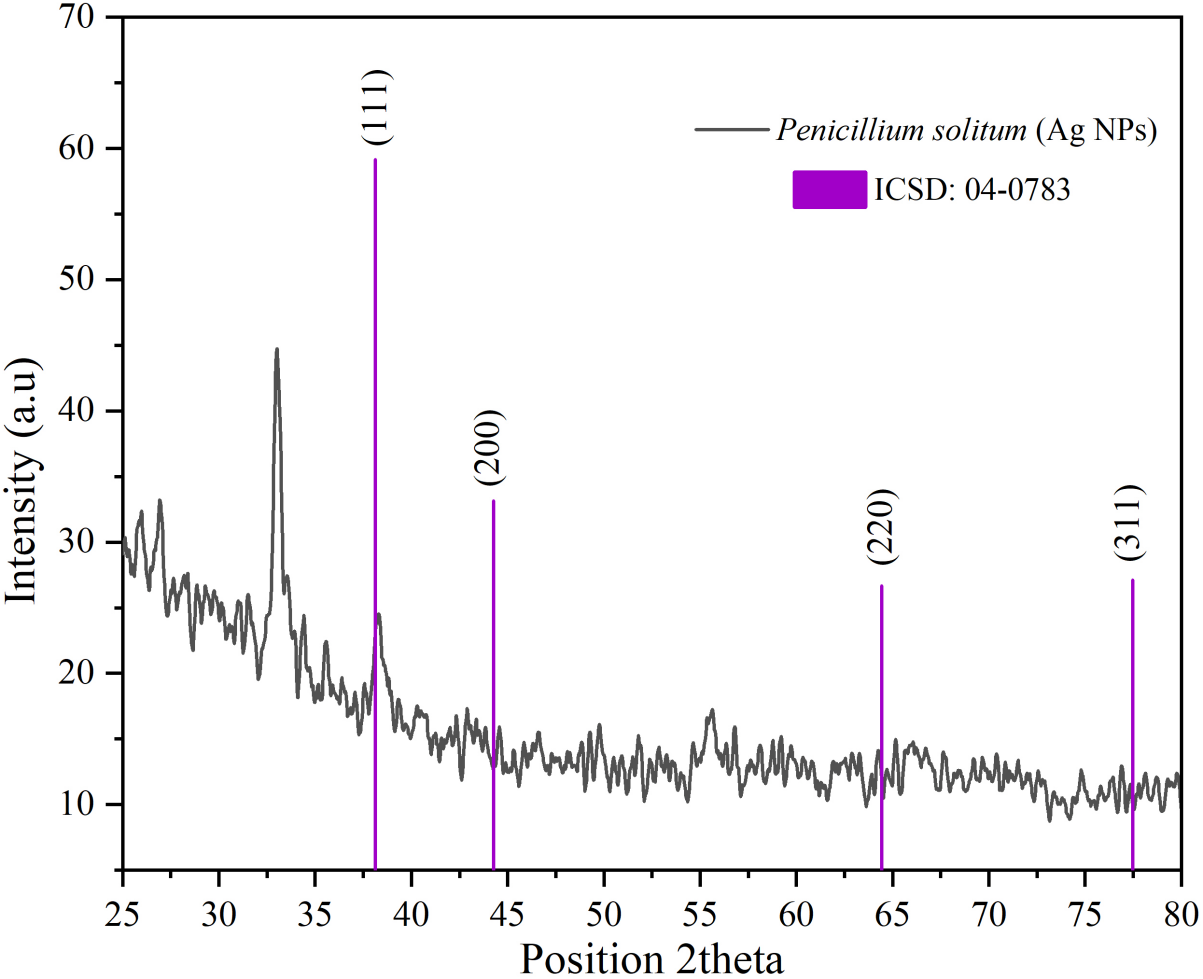
XRD pattern of Ag NPs of *Penicillium solitum*.

The diffractogram of Ag NPs is represented in **Figure 10**. The XRD pattern of synthesized Ag NPs by the reduction of silver ions with aqueous extracts of *Penicillium solitum* showed distinctive peaks in the spectrum with 2θ values ranging from 20°–80°. Indexing of the peaks was carried out by comparing with the crystal faces of Ag (ICSD No.04-0783) of face center cubic. The diffraction pattern of *Penicillium solitum* Ag NPs displayed four strong peaks at 2θ values the characteristic peaks at 36.14°, 44.26°, 64.42° and 77.44° correspond to the (111), (200), (220) and (311) structure. Four distinct diffraction peaks of silver were observed at Bragg’s planes (111, 200, 220, 311) for *Penicillium brasilianum* Ag NPs. These values represent the face-centered cubic structure of synthesized Ag NPs. A similar diffraction pattern was obtained with *Penicillium diversum* Ag NPs that showed biological components such as proteins, enzymes or metabolites in Ag NPs crystals formation (Ganachari et al., 2012; Rudrappa et al., 2023). Another study also indicated 2θ values corresponded to silver crystalline structure of *Penicillium atramentosum* KM based Ag NPs (Sarsar et al., 2015).

### 3.7 FE SEM of *Penicillium solitum* Ag NPs

**Figure 11:**
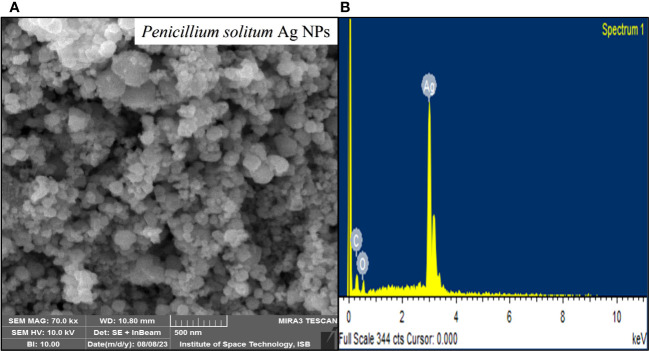
SEM images of **(A)** Ag NPs and **(B)** EDX of Ag NPs.

SEM is used to characterize the surface morphology, size, aggregation, distribution of nanoparticles where electron beam is utilized as imaging probe. It provided high-resolution image of synthesized Ag NPs at the nanometer scale, the role of bio matrix, or encapsulation of Ag NPs that contribute to nanoparticle size (Gupta et al., 2019). The microscopic structure of synthesized silver nanoparticles was found to be 18 nm. The micrograph displayed that Ag NPs were amorphous in shape. Elemental composition present in the sample was presented with the help of EDX analysis. Ag was found 95.35% by weight in synthesized Ag NPs. This showed the presence of high amount of silver in Ag NPs (**Figure 11**). EDX showed a convincing peak for oxygen with a relative mass percentage of 3.17% which confirmed its presence as a major constituent of nanoparticles. The presence of oxygen could be assigned to emission of X-rays from free amino groups from *Penicillium solitum*. Similarly, 1.35% carbon presence in EDX indicated the presence of sugars in the fungal mass extract (Dinesh et al., 2021).

### 3.8 FTIR of Ag NPs

**Figure 12:**
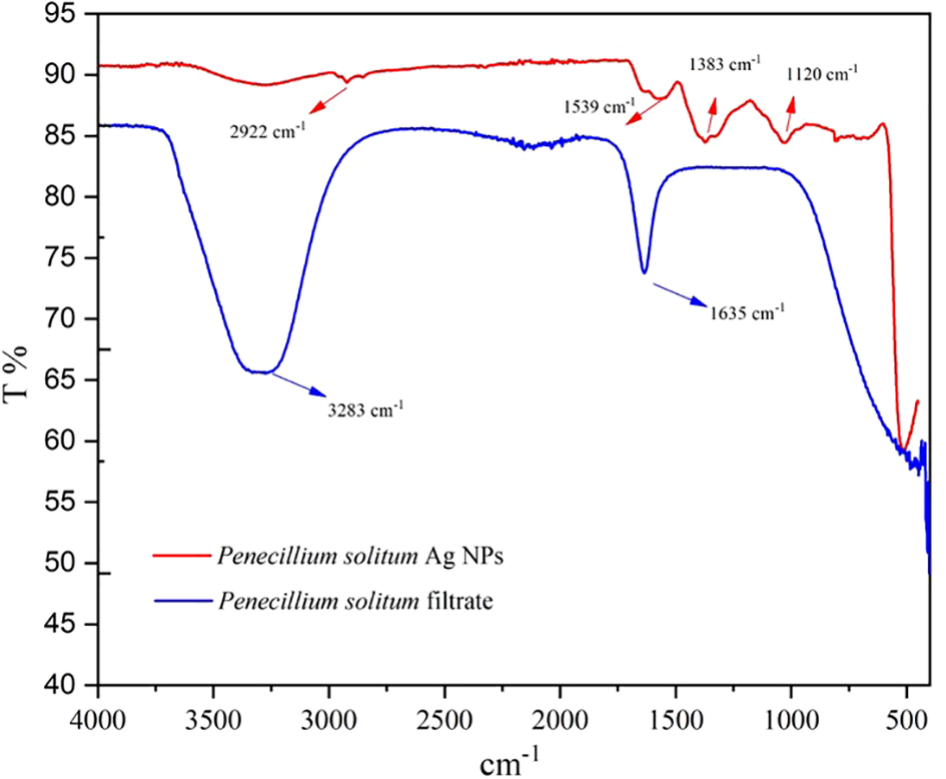
FTIR pattern of *Penicillium solitum* filtrate and Ag NPs.

The chemical composition of synthesized Ag NPs is characterized by Fourier transform infrared spectroscopy (FTIR). FTIR shows the metabolites that are involved in reduction and capping of Ag NPs, and also shows the presence of chemical residue at the surface of Ag NPs (Mourdikoudis et al., 2018). The FTIR spectrum of *Penicillium solitum* filtrate gave distinctive peaks at 3283 and 1635 cm^-1^. A broad peak at 3283 cm^-1^ showed stretching vibrations of O-H of phenolic group and N-H bend of amine group (Nassar et al., 2023). This signifies the involvement of aromatic proteins in the formation of Ag NPs. A strong peak at 1635 cm-1 corresponds to C=O of polysaccharide or N-H stretching of amide present in fungal biomass (Rudrappa et al., 2023). These functional groups in *Penicillium solitum* biomass filtrate play a critical role in reducing and capping of Ag NPs. The results of Ag NPs FTIR analysis of this study showed different stretches of bonds at various peaks of 1120, 1383, 1539, and 2922 cm^-1^. Ag NPs spectrum showed significant peak at 1,120 cm^−1^ indicating O–H stretch (Karthik et al., 2014). The band at 1383 cm^−1^ (Jyoti et al., 2016) amplifies the N-O symmetry stretching typical of the nitro compounds. The peak at 1,539 cm^−1^ corresponds to the aromatic ring of the terpenoid saponin structure (Wan Mat Khalir et al., 2020). Bandat 2922 cm^−1^ region arising from C–H stretching of aromatic compounds (Jyoti et al., 2016) was observed (**Figure 12**). During the synthesis of *Penicillium solitum* Ag NPs, biomolecules including enzymes, proteins and polysaccharides showed a strong affinity towards nanoparticles. Similar results were reported for *Penicillium brasilianum* and *Penicillium verhagenii* where amino acids, sugars and other biomolecules from fungal filtrate resulted in nanoparticles synthesis (Nassar et al., 2023; Rudrappa et al., 2023). Additionally, fungi-based metabolites provided electrostatic attraction between the nanoparticles and the biomolecules thus enhancing the formation of stable Ag NPs (Gupta et al., 2023).

### 3.9 Antifungal and antibacterial activity of Ag NPs

**Figure 13:**
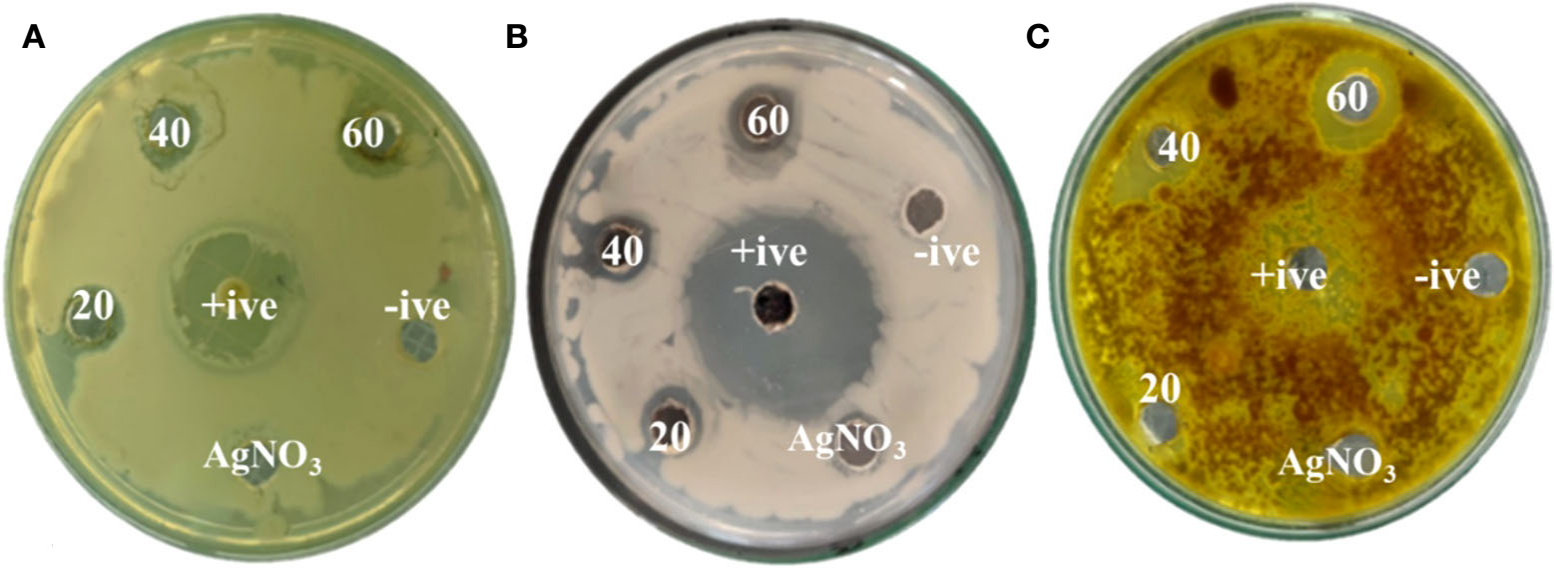
Zones of inhibition with different concentrations of *Penicillium solitum* Ag NPs (ug/mL). **(A)** *Pseudomonas syringae* DC3000 **(B)** *Streptomyces scabies* **(C)** *Aspergillus flavus*.

**Figure 14:**
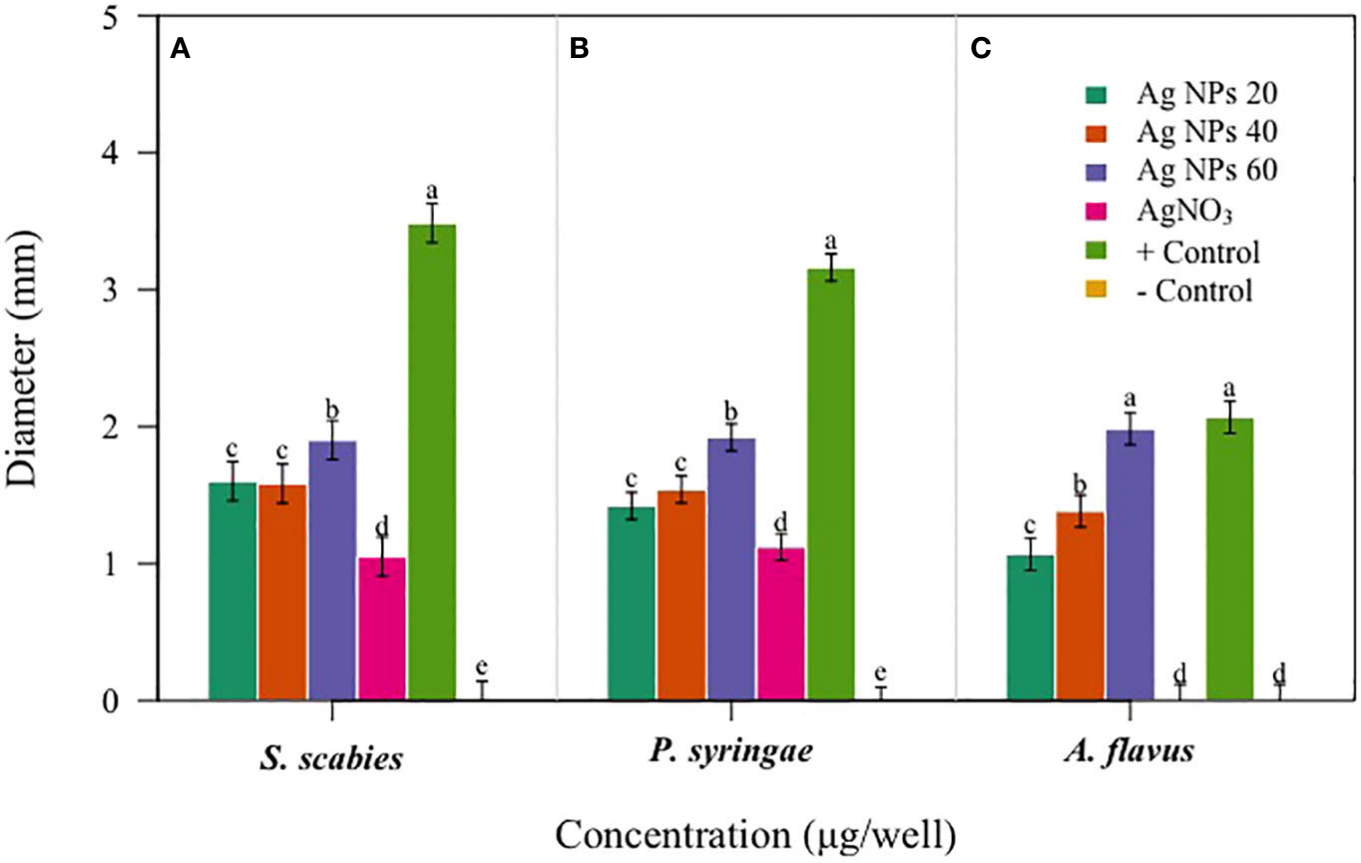
Antibacterial activity of *Penicillium solitum* Ag NPs. Bacterial and fungal zone of inhibition with Ag NPs at 20, 40 and 60 ug/ul Ag NPs **(A)** *Streptomyces scabies* **(B)** *Pseudomonas syringae* **(C)** *Aspergillus flavus*. Data is represented as means ± SE from 3 replicates. Different letters represent significant differences at p ≤ 0.05 Fisher LSD.

**Figure 15:**
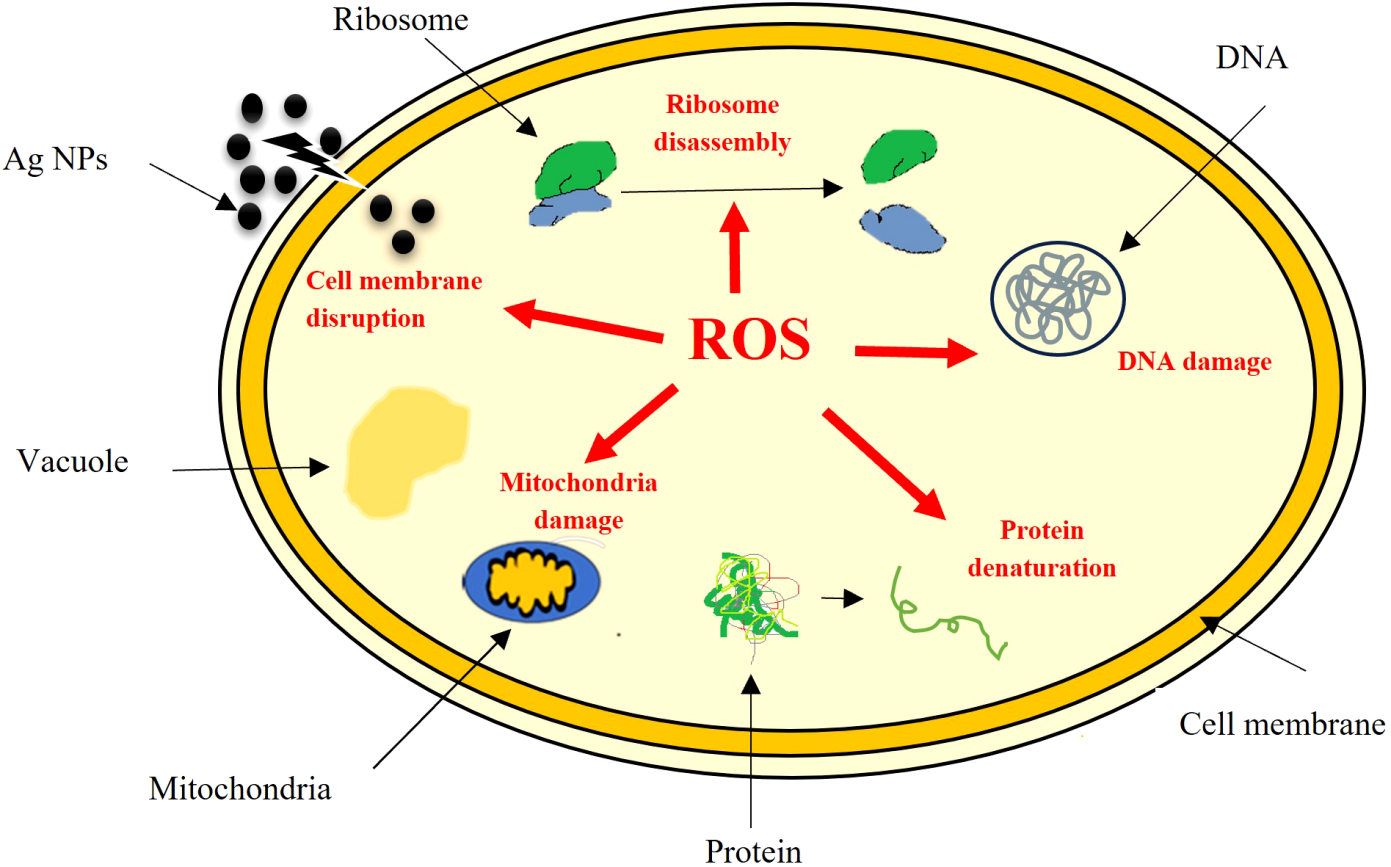
Schematic representation of antimicrobial mechanism of *Penicillium solitum* Ag NPs.

The potential impact of fungal nanoparticles against plant pathogens is comparatively higher than other microbial NPs due to their greater production of bioactive molecules involved in the synthesis of green NPs. It is previously reported that *Fusarium oxysporum*, *Rhizopus stolonifer* and *Penicillium fellutanum* fungal strains were successfully used for the synthesis of silver NPs (Nanda et al., 2015; Ali et al., 2020). To check the antimicrobial potential of *Penicillium solitum*, the Ag NPs synthesized was subjected to antimicrobial activity against different phyto-pathogens including *Aspergillus flavus, Streptomyces scabies* and *Pseudomonas syringe* DC3000. The maximum zone of inhibition was significant with 1.90, 1.92 and 1.98 diameter (mm) respectively with 60 ug/ul compared to kanamycin and Voriconazole (60ug/ul). The Ag NPs of *Penicillium solitum* showed strong antimicrobial activity against *Streptomyces scabies* (gram-positive), *Pseudomonas syringae* DC3000 (gram-negative) and *Aspergillus flavus* based on one-way ANOVA with LSD at 0.05 level of significance (**Figures 13**, **14**; R Core Team, 2023). It was found that NADPH-mediated nitrate reductase enzymes are responsible for the formation of Ag NPs for the reduction of silver ions from fungal species (Kumar et al., 2007). Biomolecules of fungal origin such as proteins and amino acid residues provide amino groups, carboxyl groups or hydroxyl groups having nucleophilic affinity that play an essential role in Ag NPs formation and enhanced antimicrobial activity (Elgorban et al., 2016). Both gram-positive and gram-negative bacteria are composed of an outer layer of peptidoglycans, however gram-negative bacteria contain an extra layer of lipopolysaccharides. Similarly, fungus cell wall is mainly composed of complex polysaccharides while its cell membrane is composed of ergosterols, fatty acids or glycophospholipids. Biogenic Ag NPs penetrate fungus and bacteria cell wall and adhere to cell membrane. These Ag NPs move inside the cell and disrupt metabolic and cellular processes including DNA degradation (Shahzadi et al., 2022). The key mechanism of Ag NPs against bacteria and fungus includes production of reactive oxygen species (ROS) such as hydroxyl radical, superoxide and hydrogen peroxide. Excessive ROS causes protein dysfunction, lipid peroxidation and DNA damage, finally causing cell apoptosis (**Figure 15**; Yousaf et al., 2022; Rudrappa et al., 2023). Based on this, synthesized Ag NPs from *Aspergillus versicolor* showed strong activity against *Sclerotinia sclerotiorum* and *Botrytis cinerea* in strawberry plants. Similar results were obtained with *Aspergillus niger* Ag NPs against *Penicillin digitatum*, *Aspergillus flavus*, and *Fusarium oxysporum* isolated from different crop species (Al-Zubaidi et al., 2019). This indicates a step forward towards nano-based fungicides/bactericides against phyto-pathogens.

## 4 Conclusion

In the current study, *Penicillium solitum* was isolated from diseased potato tubers for the first time in Pakistan. Ag NPs were synthesized using *Penicillium solitum* aqueous extract. Ag NPs showed UV peak at 410 nm. XRD and SEM confirmed the face cubic crystal with Ag NPs of 18 nm. FTIR showed biogenic Ag NPs formed based on fungal extract. Significant antimicrobial activity was found against plant pathogens *Aspergillus flavus*, *Streptomyces scabies* and *Pseudomonas syringae*. This report shows for the first time that *Penicillium solitum* based Ag NPs could be used as a potential drug against harmful pathogens. This further requires secondary metabolites pathway elucidation for synthesis of Ag NPs for their successful implementation in agriculture practices.

## Data availability statement

The datasets presented in this study can be found in online repositories. The names of the repository/repositories and accession number(s) can be found in the article/**Supplementary Material**.

## Author contributions

SS: Investigation, Methodology, Writing – original draft. XS: Methodology, Data curation, Formal Analysis, Writing – review & editing. SB: Data curation, Formal Analysis, Methodology, Software, Writing – original draft. HZ: Validation, Writing – review & editing. BI: Writing – review & editing, Data curation, Formal Analysis. IS: Writing – review & editing. ZM: Writing – review & editing. SN: Writing – review & editing. MS: Writing – review & editing. AB: Writing – review & editing, Conceptualization.
