# Supplementary material for: First identification of potato tuber rot caused by Penicillium solitum, its silver nanoparticles synthesis, characterization and use against harmful pathogens
Source: Front Plant Sci. 2023 Oct 19;14:1255480. doi: 10.3389/fpls.2023.1255480 (PMC10620797; doi:10.3389/fpls.2023.1255480)
Supplement: Supplementary file 1 [file DataSheet_1.docx]

>MN565908.1 Penicillium citrinum isolate MT14 internal transcribed spacer 1, partial sequence; 5.8S ribosomal RNA gene and internal transcribed spacer 2, complete sequence; and large subunit ribosomal RNA gene, partial sequence

GCGTAAGGTACTGCGGAGACATTACCGAGTGAGGGCCCTTTGGGTCCAACCTCCCACCCGTGTTTATTTTACCTTGTTGCTTCGGCGGGCCCGCCTTTACTGGCCGCCGGGGGGCTCACGCCCCCGGGCCCGCGCCCGCCGAAGACACCCCCGAACTCTGTCTGAAGATTGAAGTCTGAGTGAAAATATAAATTATTTAAAACTTTCAACAACGGATCTCTTGGTTCCGGCATCGATGAAGAACGCAGCGAAATGCGATACGTAATGTGAATTGCAAATTCAGTGAATCATCGAGTCTTTGAACGCACATTGCGCCCCCTGGTATTCCGGGGGGCATGCCTGTCCGAGCGTCATTGCTGCCCTCAAGCCCGGCTTGTGTGTTGGGCCCCGTCCTCCGATTCCGGGGGACGGGCCCGAAAGGCAGCGGCGGCACCGCGTCCGGTCCTCGAGCGTATGGGGCTTTGTCACCCGCTCTGTAGGCCCGGCCGGCGCTTGCCGATCAACCCAAATTTTTATCCAGGTTGACCTCGGATCATGTAGGGATACCCGCTGAACTTAAGCATATCAATAAGCGGAGGAAAATA

>MK567887.1 Penicillium solitum isolate JJGG-55 internal transcribed spacer 1, partial sequence; 5.8S ribosomal RNA gene and internal transcribed spacer 2, complete sequence; and large subunit ribosomal RNA gene, partial sequence

CTTAGTACTGCGGAGACATTACCGAGTGAGGGCCCTTTGGGTCCAACCTCCCACCCGTGTTTATTTTACCTTGTTGCTTCGGCGGGCCCGCCTTTACTGGCCGCCGGGGGGCTCACGCCCCCGGGCCCGCGCCCGCCGAAGACACCCCCGAACTCTGTCTGAAGATTGAAGTCTGAGTGAAAATATAAATTATTTAAAACTTTCAACAACGGATCTCTTGGTTCCGGCATCGATGAAGAACGCAGCGAAATGCGATACGTAATGTGAATTGCAAATTCAGTGAATCATCGAGTCTTTGAACGCACATTGCGCCCCCTGGTATTCCGGGGGGCATGCCTGTCCGAGCGTCATTGCTGCCCTCAAGCCCGGCTTGTGTGTTGGGCCCCGTCCTCCGATTCCGGGGGACGGGCCCGAAAGGCAGCGGCGGCACCGCGTCCGGTCCTCGAGCGTATGGGGCTTTGTCACCCGCTCTGTAGGCCCGGCCGGCGCTTGCCGATCAACCCAAATTTTTATCCAGGTTGACCTCGGATCAGGTAGGGATACCCGCTGAACTTAAGCATATCTAAACCGGGAAGGAAAAAAA

>KY817990.1 Penicillium sp. 18S ribosomal RNA gene, partial sequence; internal transcribed spacer 1, 5.8S ribosomal RNA gene, and internal transcribed spacer 2, complete sequence; and 28S ribosomal RNA gene, partial sequence

GCGTAAGGTACTGCGGAGACATTACCGAGTGAGGGCCCTTTGGGTCCAACCTCCCACCCGTGTTTATTTTACCTTGTTGCTTCGGCGGGCCCGCCTTTACTGGCCGCCGGGGGGCTCACGCCCCCGGGCCCGCGCCCGCCGAAGACACCCCCGAACTCTGTCTGAAGATTGAAGTCTGAGTGAAAATATAAATTATTTAAAACTTTCAACAACGGATCTCTTGGTTCCGGCATCGATGAAGAACGCAGCGAAATGCGATACGTAATGTGAATTGCAAATTCAGTGAATCATCGAGTCTTTGAACGCACATTGCGCCCCCTGGTATTCCGGGGGGCATGCCTGTCCGAGCGTCATTGCTGCCCTCAAGCCCGGCTTGTGTGTTGGGCCCCGTCCTCCGATTCCGGGGGACGGGCCCGAAAGGCAGCGGCGGCACCGCGTCCGGTCCTCGAGCGTATGGGGCTTTGTCACCCGCTCTGTAGGCCCGGCCGGCGCTTGCCGATCAACCCAAATTTTTATCCAGGTTGACCTCGGATCATGTAG

>KC692223.1 Penicillium polonicum strain ML329 18S ribosomal RNA gene, partial sequence; internal transcribed spacer 1, 5.8S ribosomal RNA gene, and internal transcribed spacer 2, complete sequence; and 28S ribosomal RNA gene, partial sequence

GGCTTAGCTCGTAGTGACCTGCGGAGGATCATTACCGAGTGAGGGCCCTTTGGGTCCAACCTCCCACCCGTGTTTATTTTACCTTGTTGCTTCGGCGGGCCCGCCTTTACTGGCCGCCGGGGGGCTCACGCCCCCGGGCCCGCGCCCGCCGAAGACACCCCCGAACTCTGTCTGAAGATTGAAGTCTGAGTGAAAATATAAATTATTTAAAACTTTCAACAACGGATCTCTTGGTTCCGGCATCGATGAAGAACGCAGCGAAATGCGATACGTAATGTGAATTGCAAATTCAGTGAATCATCGAGTCTTTGAACGCACATTGCGCCCCCTGGTATTCCGGGGGGCATGCCTGTCCGAGCGTCATTGCTGCCCTCAAGCCCGGCTTGTGTGTTGGGCCCCGTCCTCCGATTCCGGGGGACGGGCCCGAAAGGCAGCGGCGGCACCGCGTCCGGTCCTCGAGCGTATGGGGCTTTGTCACCCGCTCTGTAGGCCCGGCCGGCGCTTGCCGATCAACCCAAATTTTTATCCAGGTTGACCTCGGATCAGGTAGGGATACCCGCTGAACTTAAGCATATCAATAAGCGGAGGAA

>MT133771.1 Penicillium cyclopium strain UBOCC-A-119009 internal transcribed spacer 1, partial sequence; 5.8S ribosomal RNA gene and internal transcribed spacer 2, complete sequence; and large subunit ribosomal RNA gene, partial sequence

GACTGCGGAGGATCATTACCGAGTGAGGGCCCTTTGGGTCCAACCTCCCACCCGTGTTTATTTTACCTTGTTGCTTCGGCGGGCCCGCCTTTACTGGCCGCCGGGGGGCTCACGCCCCCGGGCCCGCGCCCGCCGAAGACACCTCCGAACTCTGTCTGAAGATTGAAGTCTGAGTGAAAATATAAATTATTTAAAACTTTCAACAACGGATCTCTTGGTTCCGGCATCGATGAAGAACGCAGCGAAATGCGATACGTAATGTGAATTGCAAATTCAGTGAATCATCGAGTCTTTGAACGCACATTGCGCCCCCTGGTATTCCGGGGGGCATGCCTGTCCGAGCGTCATTGCTGCCCTCAAGCACGGCTTGTGTGTTGGGCCCCGTCCTCCGATTCCGGGGGACGGGCCCGAAAGGCAGCGGCGGCACCGCGTCCGGTCCTCGAGCGTATGGGGCTTTGTCACCCGCTCTGTAGGCCCGGCCGGCGCTTGCCGATCAACCCAAATTTTTATCCAGGTTGACCTCGGATCAGGTAGGGATACCCGCTGAACTTAAGCATATCTAGG

>MF979522.1 Penicillium polonicum isolate FA_16 small subunit ribosomal RNA gene, partial sequence; internal transcribed spacer 1, 5.8S ribosomal RNA gene, and internal transcribed spacer 2, complete sequence; and large subunit ribosomal RNA gene, partial sequence

AGTAAAAAATGTAACAAGGTTTCCGTAGGTGAACCTGCGGAAGGATCATTACCGAGTGAGGGCCCTTTGGGTCCAACCTCCCCACCCGTGTTTATTTTACCTTGTTGCTTCGGCGGGCCCGCCTTTACTGGCCGCCGGGGGGCTCACGCCCCCGGGCCCGCGCCCGCCGAAGACACCCCCGAACTCTGTCTGAAGATTGAAGTCTGAGTGAAAATATAAATTATTTAAAACTTTCAACAACGGATCTCTTGGTTCCGGCATCGATGAAGAACGCAGCGAAATGCGATACGTAATGTGAATTGCAAATTCAGTGAATCATCGAGTCTTTGAACGCACATTGCGCCCCCTGGTATTCCGGGGGGCATGCCTGTCCGAGCGTCATTGCTGCCCTCAAGCCCGGCTTGTGTGTTGGGCCCCGTCCTCCGATTCCGGGGGACGGGCCCGAAAGGCAGCGGCGGCACCGCGTCCGGTCCTCGAGCGTATGGGGCTTTGTCACCCGCTCTGTAGGCCCGGCCGGCGCTTGCCGATCAACCCAAATTTTTATCCAGGTTGACCTCGGATCAGGTAGGGATACCCGCTGAACTTAAGCATATCATAAG

>KF881751.1 Penicillium sp. SCAU-F-14 18S ribosomal RNA gene, partial sequence; internal transcribed spacer 1, 5.8S ribosomal RNA gene, and internal transcribed spacer 2, complete sequence; and 28S ribosomal RNA gene, partial sequence

AGGATTTCGTAGGTGACCTGCGGAAGGACATTACCGAGTGAGGGCCCTTTGGGTCCAACCTCCCACCCGTGTTTATTTTA

CCTTGTTGCTTCGGCGGGCCCGCCTTTACTGGCCGCCGGGGGGCTCACGCCCCCGGGCCCGCGCCCGCCGAAGACACCCCCGAACTCTGTCTGAAGATTGAAGTCTGAGTGAAAATATAAATTATTTAAAACTTTCAACAACGGATCTCTTGGTTCCGGCATCGATGAAGAACGCAGCGAAATGCGATACGTAATGTGAATTGCAAATTCAGTGAATCATCGAGTCTTTGAACGCACATTGCGCCCCCTGGTATTCCGGGGGGCATGCCTGTCCGAGCGTCATTGCTGCCCTCAAGCCCGGCTTGTGTGTTGGGCCCCGTCCTCCGATTCCGGGGGACGGGCCCGAAAGGCAGCGGCGGCACCGCGTCCGGTCCTCGAGCGTATGGGGCTTTGTCACCCCTCTGTAGGCCCGGCCGGCGCTTGCCGATCAACCCAAATTTTTATCCAGGTTGACCTCGGATCAGGTAGGGATACCCGCTGAACTTAAGCATATCTAA

>MT529235.1 Penicillium polonicum clone EF_586 small subunit ribosomal RNA gene, partial sequence; internal transcribed spacer 1, 5.8S ribosomal RNA gene, and internal transcribed spacer 2, complete sequence; and large subunit ribosomal RNA gene, partial sequence

AGGAAGTAAAAGTCGTAACAAGGTTTCCGTAGGTGAACCTGCGGAAGGATCATTACCGAGTGAGGGCCCTTTGGGTCCAACCTCCCACCCGTGTTTATTTTACCTTGTTGCTTCGGCGGGCCCGCCTTTACTGGCCGCCGGGGGGCTCACGCCCCCGGGCCCGCGCCCGCCGAAGACACCCCCGAACTCTGTCTGAAGATTGAAGTCTGAGTGAAAATATAAATTATTTAAAACTTTCAACAACGGATCTCTTGGTTCCGGCATCGATGAAGAACGCAGCGAAATGCGATACGTAATGTGAATTGCAAATTCAGTGAATCATCGAGTCTTTGAACGCACATTGCGCCCCCTGGTATTCCGGGGGGCATGCCTGTCCGAGCGTCATTGCTGCCCTCAAGCCCGGCTTGTGTGTTGGGCCCCGTCCTCCGATTCCGGGGGACGGGCCCGAAAGGCAGCGGCGGCACCGCGTCCGGTCCTCGAGCGTATGGGGCTTTGTCACCCGCTCTGTAGGCCCGGCCGGCGCTTGCCGATCAACCCAAATTTTTATCCAGGTTGACCTCGGATCAGGTAGGGATACCCGCTGAACTTAAGCATAT

>MK632023.1 Penicillium polonicum isolate AS-S-25 small subunit ribosomal RNA gene, partial sequence; internal transcribed spacer 1, 5.8S ribosomal RNA gene, and internal transcribed spacer 2, complete sequence; and large subunit ribosomal RNA gene, partial sequence

CTTTCCGTAGGGGGTACCTGCGGAAGGATCATTACCGAGTGAGGGCCCTTTGGGTCCAACCTCCCACCCGTGTTTATTTTACCTTGTTGCTTCGGCGGGCCCGCCTTTACTGGCCGCCGGGGGGCTCACGCCCCCGGGCCCGCGCCCGCCGAAGACACCCCCGAACTCTGTCTGAAGATTGAAGTCTGAGTGAAAATATAAATTATTTAAAACTTTCAACAACGGATCTCTTGGTTCCGGCATCGATGAAGAACGCAGCGAAATGCGATACGTAATGTGAATTGCAAATTCAGTGAATCATCGAGTCTTTGAACGCACATTGCGCCCCCTGGTATTCCGGGGGGCATGCCTGTCCGAGCGTCATTGCTGCCCTCAAGCCCGGCTTGTGTGTTGGGCCCCGTCCTCCGATTCCGGGGGACGGGCCCGAAAGGCAGCGGCGGCACCGCGTCCGGTCCTCGAGCGTATGGGGCTTTGTCACCCGCTCTGTAGGCCCGGCCGGCGCTTGCCGATCAACCCAAATTTTTATCCAGGTTGACCTCGGATCAGGTAGGGATACCCGCTGAACTTAAGCATATCAATAAGCGGAGATTCCATAGTCAGGGC

>KJ676451.1 Penicillium lapidosum strain ssw 18S ribosomal RNA gene, partial sequence; internal transcribed spacer 1, 5.8S ribosomal RNA gene, and internal transcribed spacer 2, complete sequence; and 28S ribosomal RNA gene, partial sequence

TTCCGATGGTGAACCTGCGGAAGGATCATTACCGAGTGAGGGCCCTTTGGGTCCAACCTCCCACCCGTGTTTATTTTACCTTGTTGCTTCGGCGGGCCCGCCTTTACTGGCCGCCGGGGGGCTCACGCCCCCGGGCCCGCGCCCGCCGAAGACACCCCCGAACTCTGTCTGAAGATTGAAGTCTGAGTGAAAATATAAATTATTTAAAACTTTCAACAACGGATCTCTTGGTTCCGGCATCGATGAAGAACGCAGCGAAATGCGATACGTAATGTGAATTGCAAATTCAGTGAATCATCGAGTCTTTGAACGCACATTGCGCCCCCTGGTATTCCGGGGGGCATGCCTGTCCGAGCGTCATTGCTGCCCTCAAGCCCGGCTTGTGTGTTGGGCCCCGTCCTCCGATTCCGGGGGACGGGCCCGAAAGGCAGCGGCGGCACCGCGTCCGGTCCTCGAGCGTATGGGGCTTTGTCACCCGCTCTGTAGGCCCGGCCGGCGCTTGCCGATCAACCCAAATTTTTATCCAGGTTGACCTCGGATCAGGTAGGGATACCCGCTGAACTTAAGCATATCAATAAGCGGAGGA

>ON310860

TCTTAGTTCCTGCGGTAAGACATTTACCGAGTGAGGGCCCTTTGGGTCCAACCTCCCCACCCGTGTTTATTTTACCTTGTTGCTTCGGCGGGCCCGCCTTTACTGGCCGCCGGGGGGCTCACGCCCCCGGGCCCGCGCCCGCCGAAGACACCCCCGAACTCTGTCTGAAGATTGAAGTCTGAGTGAAAATATAAATTATTTAAAACTTTCAACAACGGATCTCTTGGTTCCGGCATCGATGAAGAACCGCAGCGAAAATTTTTTTTTTTT

>ON310801

TTGGTGACTGCGGAGACATTACCGAGTGAGGGCCCTTTGGGTCCAACCTCCCACCCGTGTTTATTTTACCTTGTTGCTTCGGCGGGCCCGCCTTTACTGGCCGCCGGGGGGCTCACGCCCCCGGGCCCGCGCCCGCCGAAGACACCCCCGAACTCTGTCTGAAGATTGAAGTCTGAGTGAAAATATAAATTATTTAAAACTTTCAACAACGGATCTCTTGGTTCCGGCATCGATGAAGACGCAGCGAAAA

>ON310863

TTGGTACTGCGGAGACATTACCGAGTGAGGGCCCTTTGGGTCCAACCTCCCACCCGTGTTTATTTTACCTTGTTGCTTCGGCGGGCCCGCCTTTACTGGCCGCCGGGGGGCTCACGCCCCCGGGCCCGCGCCCGCCGAAGACACCCCCGAACTCTGTCTGAAGATTGAAGTCTGAGTGAAAATATAAATTATTTAAAACTTTCAACAACGGATCTCTTGGTTCCGGCATCGATGAAGACGCAGCGAAAA

>ON311148

TAGGGTACTGCGGAGACATTACCGAGTGAGGGCCCTTTGGGTCCAACCTCCCACCCGTGTTTATTTTACCTTGTTGCTTCGGCGGGCCCGCCTTTACTGGCCGCCGGGGGGCTCACGCCCCCGGGCCCGCGCCCGCCGAAGACACCCCCGAACTCTGTCTGAAGATTGAAGTCTGAGTGAAAATATAAATTATTTAAAACTTTCAACAACGGATCTCTTGGTTCCGGCATCGATGAAGACGCAGCGAAAA

>ON318976

TTGGGACTGCGGAGACATTACCGAGTGAGGGCCCTTTGGGTCCAACCTCCCACCCGTGTTTATTTTACCTTGTTGCTTCGGCGGGCCCGCCTTTACTGGCCGCCGGGGGGCTCACGCCCCCGGGCCCGCGCCCGCCGAAGACACCCCCGAACTCTGTCTGAAGATTGAAGTCTGAGTGAAAATATAAATTATTTAAAACTTTCAACAACGGATCTCTTGGTTCCGGCATCGATGAAGACGCAGCGAAAA

>ON310800

TTGCGACTGCGGAGACATTACCGAGTGAGGGCCCTTTGGGTCCAACCTCCCACCCGTGTTTATTTTACCTTGTTGCTTCGGCGGGCCCGCCTTTACTGGCCGCCGGGGGGCTCACGCCCCCGGGCCCGCGCCCGCCGAAGACACCCCCGAACTCTGTCTGAAGATTGAAGTCTGAGTGAAAATATAAATTATTTAAAACTTTCAACAACGGATCTCTTGGTTCCGGCATCGATGAAGACGCAGCGAAAA

>ON310830

TAGGGACCTGCGGAGACATTACCGAGTGAGGGCCCTTTGGGTCCAACCTCCCACCCGTGTTTATTTTACCTTGTTGCTTCGGCGGGCCCGCCTTTACTGGCCGCCGGGGGGCTCACGCCCCCGGGCCCGCGCCCGCCGAAGACACCCCCGAACTCTGTCTGAAGATTGAAGTCTGAGTGAAAATATAAATTATTTAAAACTTTCAACAACGGATCTCTTGGTTCCGGCATCGATGAAGAACGCAGCGAAAA

>ON310858

TTGGTAACTGCGGAGACATTACCGAGTGAGGGCCCTTTGGGTCCAACCTCCCACCCGTGTTTATTTTACCTTGTTGCTTCGGCGGGCCCGCCTTTACTGGCCGCCGGGGGGCTCACGCCCCCGGGCCCGCGCCCGCCGAAGACACCCCCGAACTCTGTCTGAAGATTGAAGTCTGAGTGAAAATATAAATTATTTAAAACTTTCAACAACGGATCTCTTGGTTCCGGCATCGATGAAGACGCAGCGAAAA

>ON307317

TTGCTGACTGCGGAGACATTACCGAGTGAGGGCCCTTTGGGTCCAACCTCCCACCCGTGTTTATTTTACCTTGTTGCTTCGGCGGGCCCGCCTTTACTGGCCGCCGGGGGGCTCACGCCCCCGGGCCCGCGCCCGCCGAAGACACCCCCGAACTCTGTCTGAAGATTGAAGTCTGAGTGAAAATATAAATTATTTAAAACTTTCAACAACGGATCTCTTGGTTCCGGCATCGATGAAGACGCAGCGAAAA

>ON310859

TATGGGGAACTGCGGAGACATTACCGAGTGAGGGCCCTTTGGGTCCAACCTCCCACCCGTGTTTATTTTACCTTGTTGCTTCGGCGGGCCCGCCTTTACTGGCCGCCGGGGGGCTCACGCCCCCGGGCCCGCGCCCGCCGAAGACACCCCCGAACTCTGTCTGAAGATTGAAGTCTGAGTGAAAATATAAATTATTTAAAACTTTCAACAACGGATCTCTTGGTTCCGGCATCGATGAAGAACGCAGCGAAA

>ON307475

TATGGTGACTGCGGAGACATTACCGAGTGAGGGCCCTTTGGGTCCACCTCCCACCCGTGTTTATTTTACCTTGTTGCTTCGGCGGGCCCGCCTTTACTGGCCGCCGGGGGGCTCACGCCCCCGGGCCCGCGCCCGCCGAAGACACCCCCGAACTCTGTCTGAAGATTGAAGTCTGAGTGAAAATATAAATTATTTAAAACTTTCAACAACGGATCTCTTGGTTCCGGCATCGATGAACACGCAGCGAAAATGAAGAACGCAGCGAAA

>ON352773

TAAGCGACTGCGGAGACATTACCGAGTGAGGGCCCTTTGGGTCCAACCTCCCACCCGTGTTTATTTTACCTTGTTGCTTCGGCGGGCCCGCCTTTACTGGCCGCCGGGGGGCTCACGCCCCCGGGCCCGCGCCCGCCGAAGACACCCCCGAACTCTGTCTGAAGATTGAAGTCTGAGTGAAAATATAAATTATTTAAAACTTTCAACAACGGATCTCTTGGTTCCGGCATCGATGAAGACGCAGCGAAAACGCAGCGAAA

>ON311281

TTTGGTGAACCTGCGGAGACATTACCGAGTGAGGGCCCTTTGGGTCCAACCTCCCACCCGTGTTTATTTTACCTTGTTGCTTCGGCGGGCCCGCCTTTACTGGCCGCCGGGGGGCTCACGCCCCCGGGCCCGCGCCCGCCGAAGACACCCCCGAACTCTGTCTGAAGATTGAAGTCTGAGTGAAAATATAAATTATTTAAAACTTTCAACAACGGATCTCTTGGTTCCGGCATCGATGAAGAACGCAGCGAAAA

>Penicillium glabrum CBS 125543 ITS region; from TYPE material

AAGGATCATTACTGAGTGAGGGCCCTCTGGGTCCAACCTCCCACCCGTGTTTATTGTACCTTGTTGCTTCGGTGCGCCCGCCTCACGGCCGCCGGGGGGCTTCTGCCCCCGGGTCCGCGCGCACCGGAGACACTATTGAACTCTGTCTGAAGATTGCAGTCTGAGCATAAACTAAATAAGTTAAAACTTTCAACAACGGATCTCTTGGTTCCGGCATCGATGAAGAACGCAGCGAAATGCGATAACTAATGTGAATTGCAGAATTCAGTGAATCATCGAGTCTTTGAACGCACATTGCGCCCCCTGGTATTCCGGGGGGCATGCCTGTCCGAGCGTCATTGCTGCCCTCAAGCACGGCTTGTGTGTTGGGCTCCGTCCCCCCGGGGACGGGTCCGAAAGGCAGCGGCGGCACCGAGTCCGGTCCTCGAGCGTATGGGGCTTTGTCACCCGCTCTGTAGGCCCGGCCGGCGCCAGCCGACAACCAATCATCCTTTTTTCAGGTTGACCTCGGATCAGGTAGGGATACCCGCTGAACTTAAGCATATCAATAA
